# Supplementary material for: Cyclosporine A-resistant CAR-T cells mediate antitumour immunity in the presence of allogeneic cells
Source: Nat Commun. 2023 Dec 20;14:8491. doi: 10.1038/s41467-023-44176-0 (PMC10733396; doi:10.1038/s41467-023-44176-0)
Supplement: Supplementary file 1 — Supplementary Information [file 41467_2023_44176_MOESM1_ESM.pdf]

Cyclosporine A-resistant CAR-T cells mediate antitumour immunity in the presence of allogeneic cells

Yixi Zhang, Hongyu Fang, Guocan Wang, Guangxun Yuan, Ruoyu Dong, Jijun Luo, Yu Lyu, Yajie Wang, Peng Li, Chun Zhou, Weiwei Yin, Haowen Xiao, Jie Sun, Xun Zeng

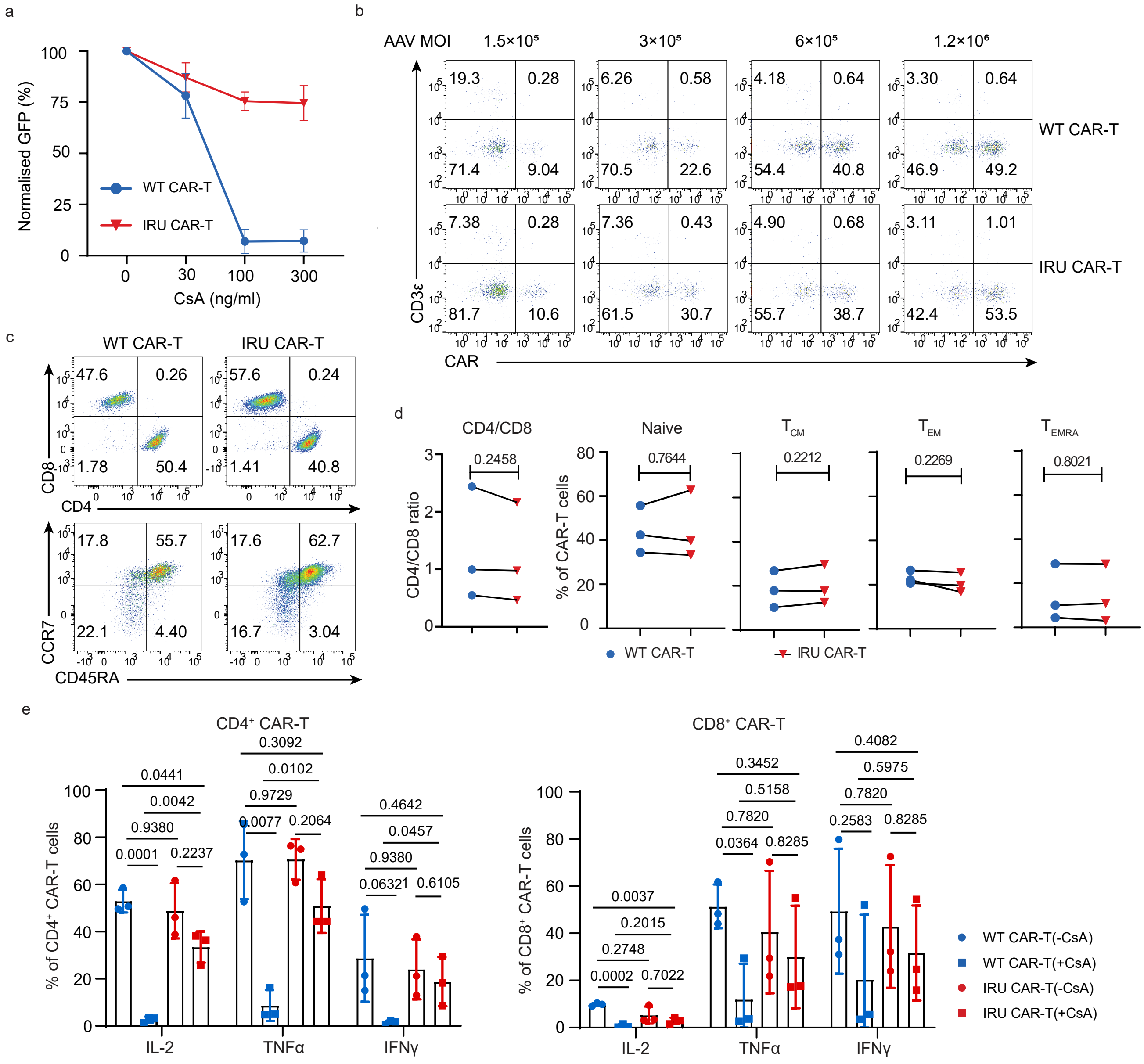

Supplementary Fig. 1 | IRU CAR-T cells retained effector functions and had the similar starting status of WT CAR-T cells. **a**, Jurkat-NFAT-GFP cells transduced with WT/IRU CARs were stimulated with Nalm6-FFluc-GFP in the context of dose-escalated CsA (30, 100, 300 ng/ml). The GFP percentages represented the activation of NFAT signaling (n = 3 independent experiments). **b**, Representative flow plots of WT/IRU CAR-T cells 4 days after transfected by AAV6 with escalated multiplicity of infection (MOI). **c,d,** representative flow plots (c) and phenotypes (d) of WT/IRU CAR-T cells before *in vitro* assays and adoptive transfer (n = 3 biologically independent samples). **e**, percentage of CD4+ (left) and CD8+ (right) CAR-T cells that expressed cytokines upon the stimulation of Nalm6-FFluc-GFP (n=3 biologically independent samples). All data are means±s.d, P values were determined by two-tailed paired t test (**d**) or Multiple t test adjusted by the Holm-Sidak method (**a**,**e**). WT CAR-T (-CsA) as blue circle, WT CAR-T (+CsA) as blue square, IRU CAR-T (-CsA) as red circle and IRU CAR-T (+CsA) as red square.

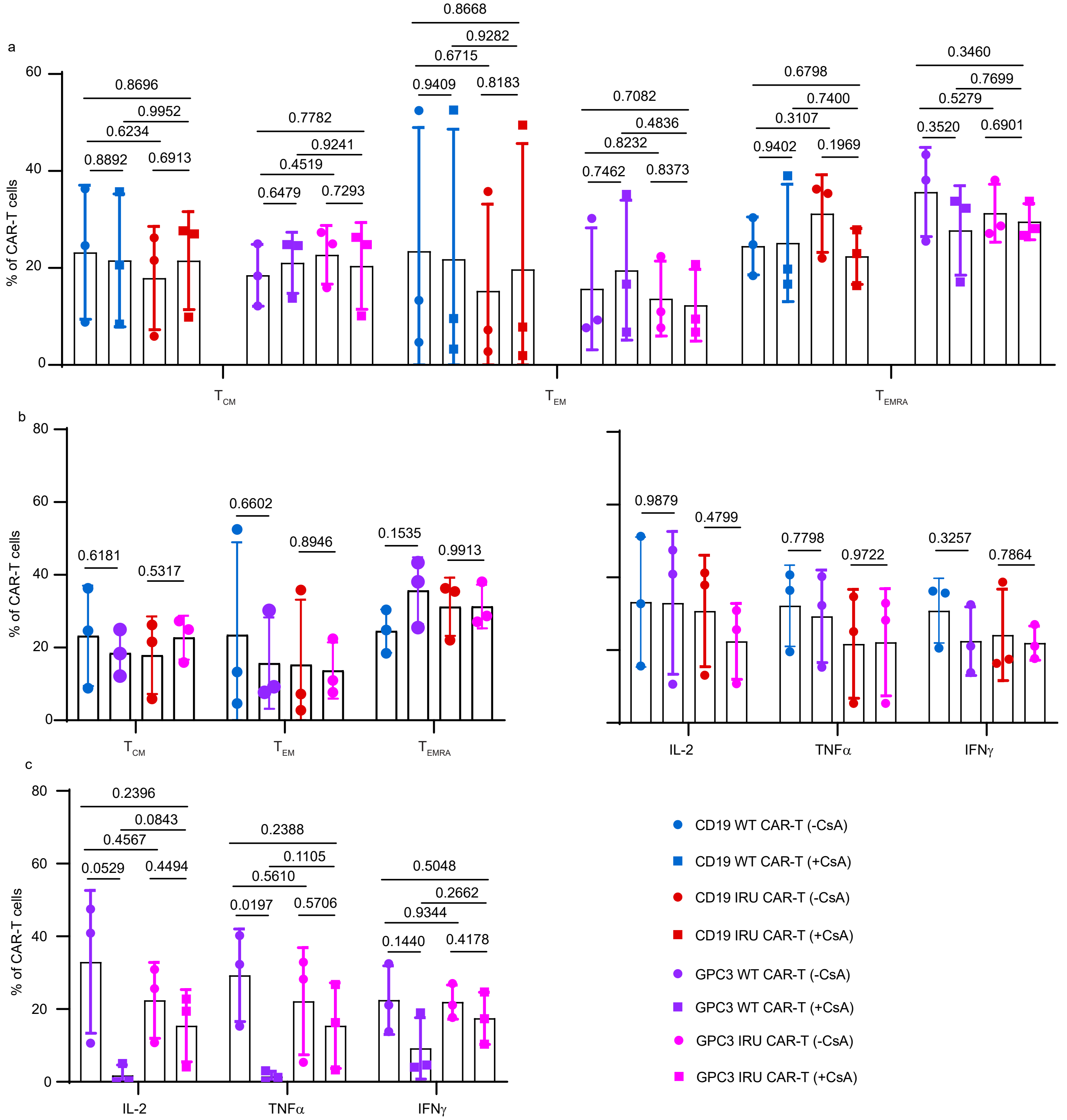

Supplementary Fig. 2 | Different CAR-T may have different phenotypes, but with similar cytokine production pattern. a, The phenotype of CD19/GPC3 WT/IRU CAR-T cells with/without CsA, with the stimulation of Nalm6/HepG2 cell line (n=3 biologically independent samples). **b**, The phenotype and cytokine comparison between CD19/GPC3 WT/IRU CAR-T cells in the absence of CsA. **c**, The cytokine production of GPC3 WT/IRU CAR-T cells with/without CsA (n=3 biologically independent samples). All data are means±s.d. P values were determined by multiple t test adjusted by the Holm-Sidak method (**a-c**). WT CAR-T (-CsA) as blue circle, WT CAR-T (+CsA) as blue square, IRU CAR-T (-CsA) as red circle and IRU CAR-T (+CsA) as red square.

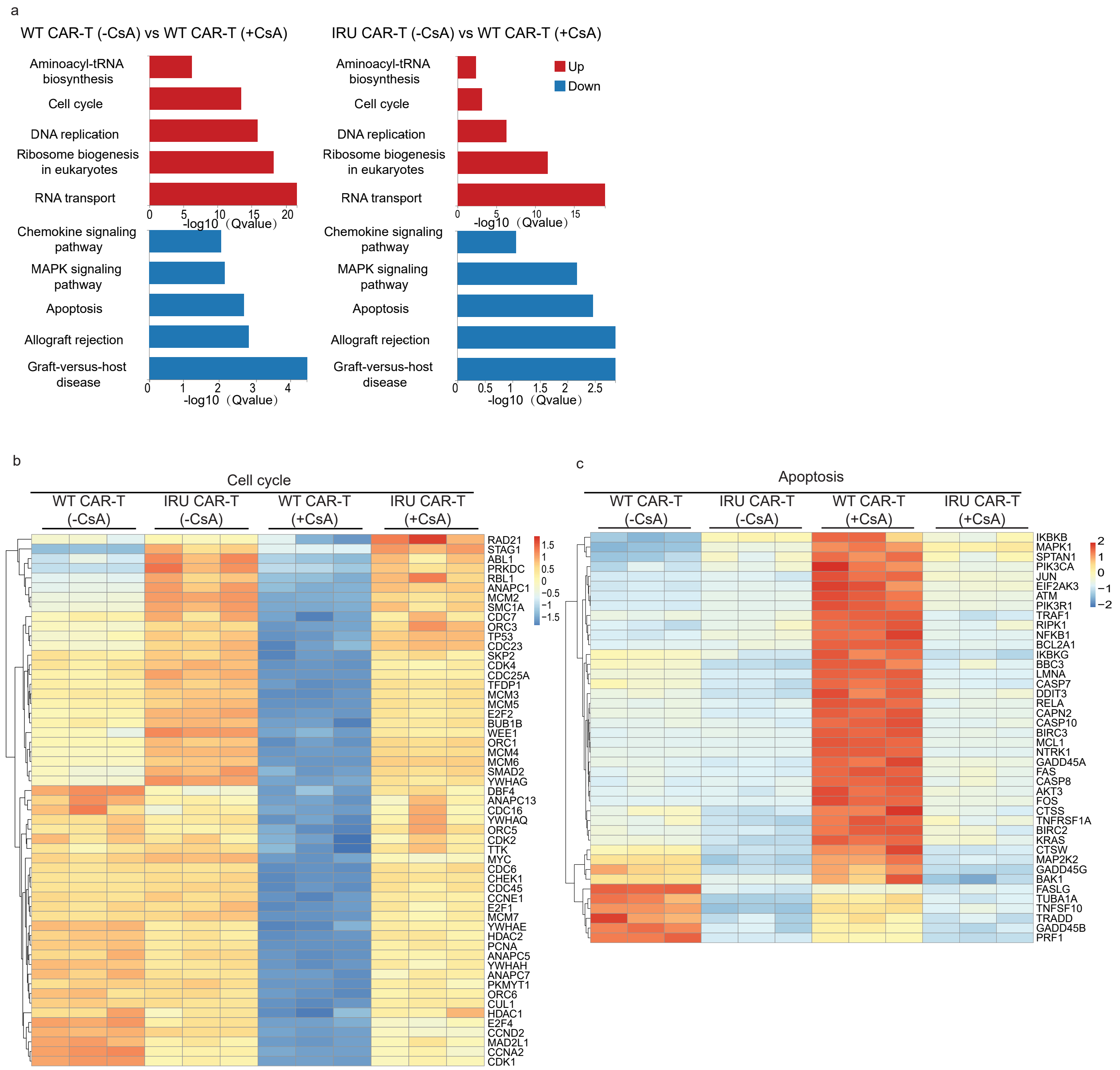

Supplementary Fig. 3 | The transcriptional signatures IRU CAR-T cells in the presence of CsA *in vitro*. a, GSEA showed the up- and down-regulated gene sets between WT CAR-T (+CsA) and WT/IRU CAR-T (-CsA) groups (n=3 independent experiments). **b,c,** Heatmap demonstrated the differences of cytotoxic and NFAT-regulated molecules among the WT/IRU CAR-T cells with/ without CsA (n=3 independent experiments).

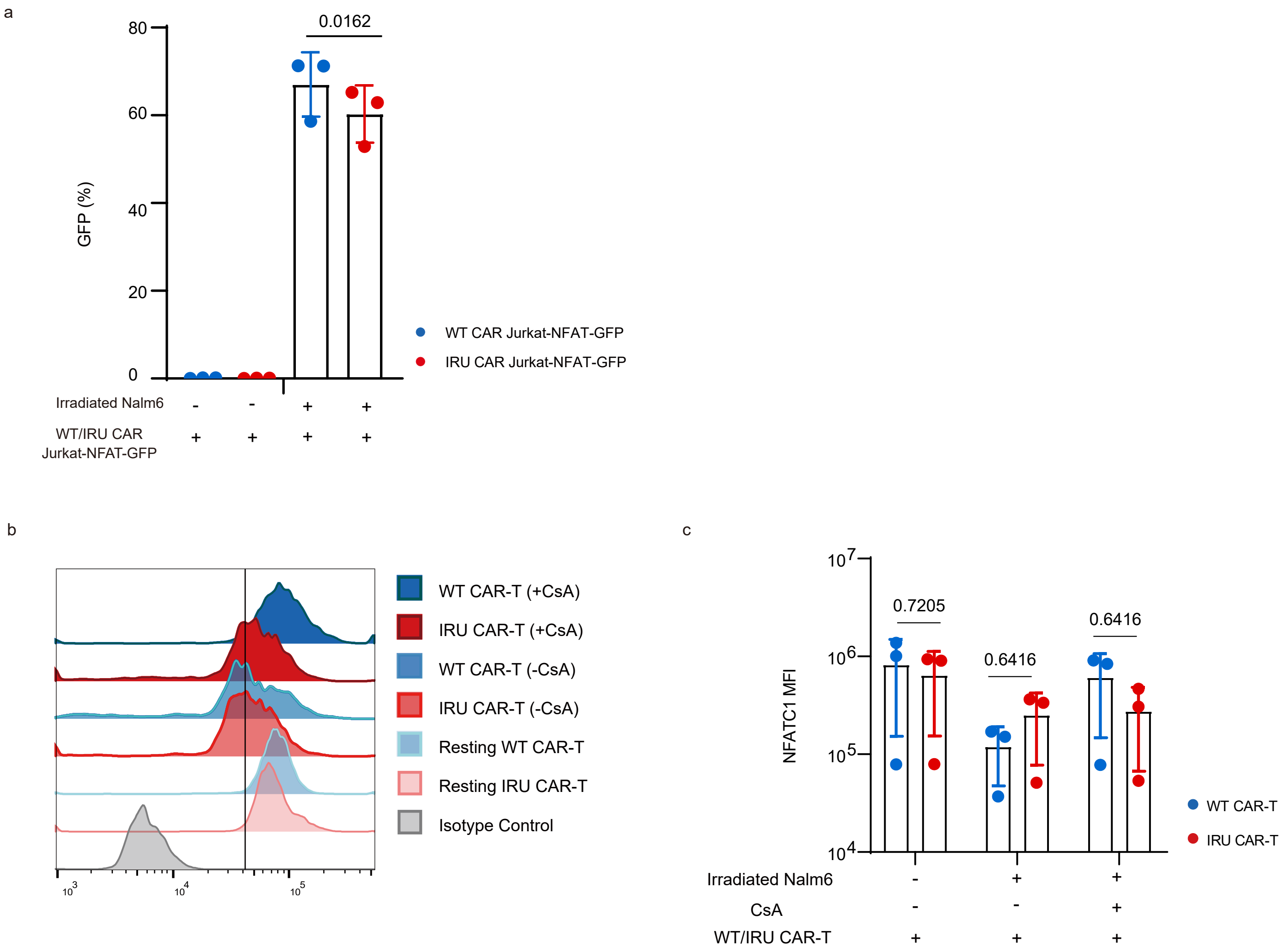

Supplementary Fig. 4 | WT and mCNA have different efficiencies of dephosphorylation of NFAT signal. a, GFP+ WT CAR-T-Jurkat-NFAT-GFP cells were significantly higher than GFP+ IRU CAR-T-Jurkat-NFAT-GFP cells, upon the stimulation of Nalm6 (n=3 independent experiments). **b,c,** representative histogram (**b**) and bar graph of MFI (**c**) of the phosphorylated NFATc1 expression in primary WT/IRU CAR-T cells (n=3 biologically independent samples). All data are means±s.d. P values were determined by Multiple t test adjusted by the Holm-Sidak method (**a,c**). WT CAR-T or WT CAR-T Jurkat-NFAT-GFP as blue circle, IRU CAR-T or IRU CAR-T Jurkat-NFAT-GFP as blue circle.

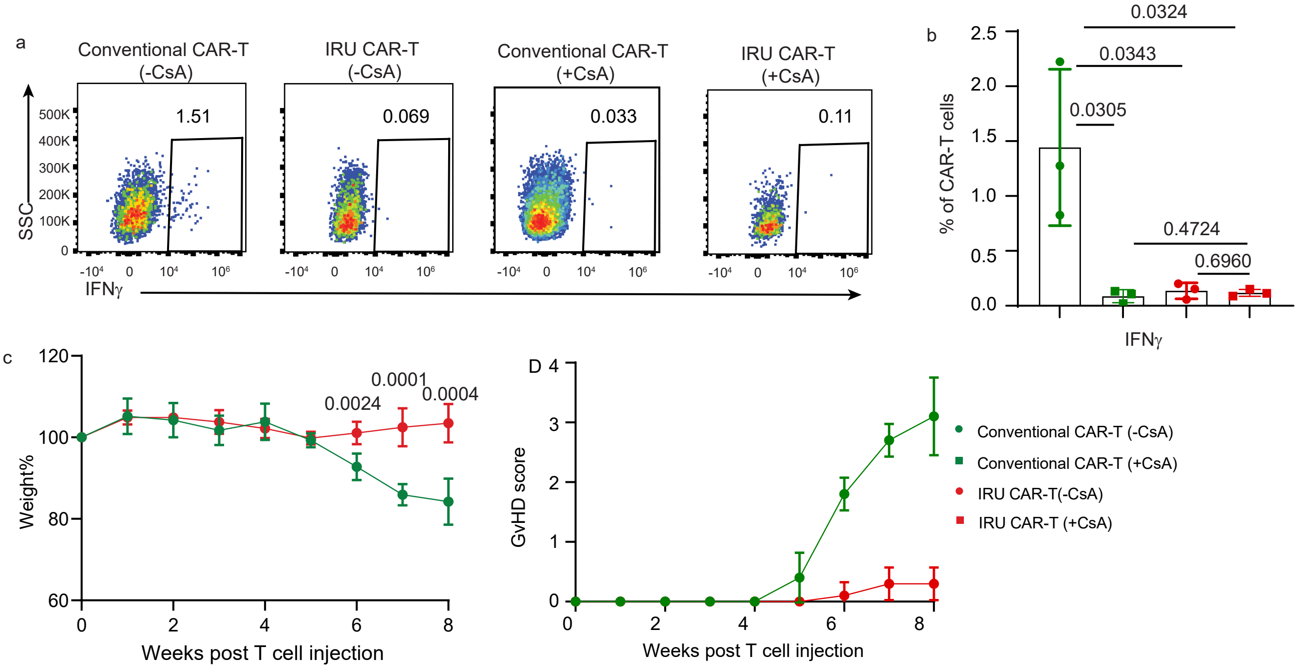

Supplementary Fig. 5 | GvHD assessment of IRU CAR-T cells *in vitro* and *in vivo*. **a, b**, Conventional/IRU CAR-T cells were cocultured with recipient PBMCs for 4 days. The IFNγ producing CAR-T cells were detected by FACS. Conventional CAR-T cells have functional *TRAC* locus (made by lentivirus infection). Representative FACS flow plots (**a**) and bar graph (**b**) of the frequency of IFNγ producing CAR-T cells from conventional/IRU CAR-T cells (n=3 biologically independent samples). **c,d**, Conventional/IRU CAR-T cells were injected into NSG mice. The weight (**c**) and GvHD score (**d**) of each mouse was assessed every week (n=5 animals). All data are means±s.d. P values were determined by Multiple t test adjusted by the Holm-Sidak method (**a**,**c**). Conventional CAR-T (-CsA) as green circle, Conventional CAR-T (+CsA) as green square, IRU CAR-T (-CsA) as red circle and IRU CAR-T (+CsA) as red square.

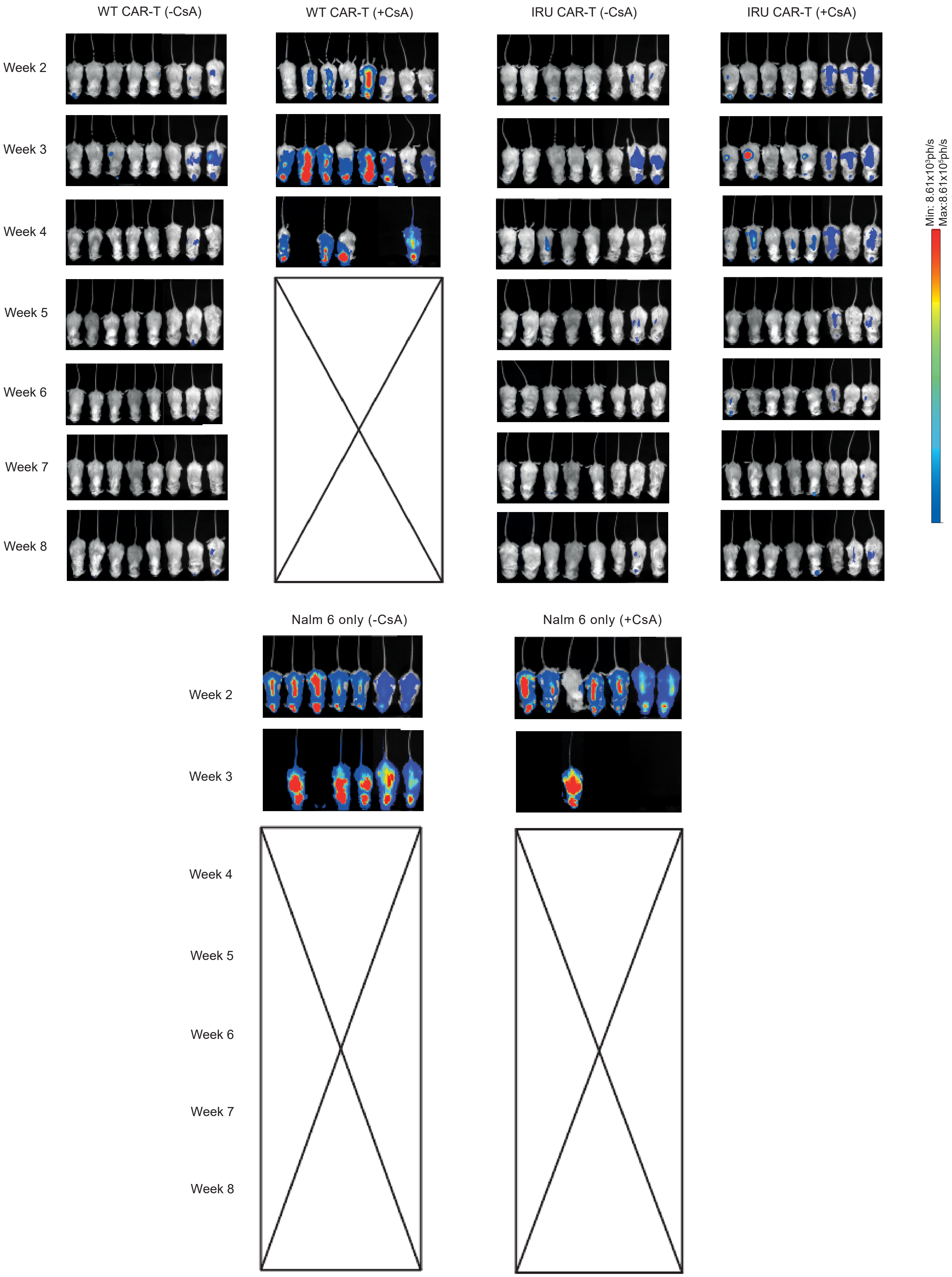

Supplementary Fig. 6 | Light emission detection showed the tumor progression in mice treated without or with CAR-T cells (WT/IRU CAR, ±CsA) (n=8 animals, 2 batches).

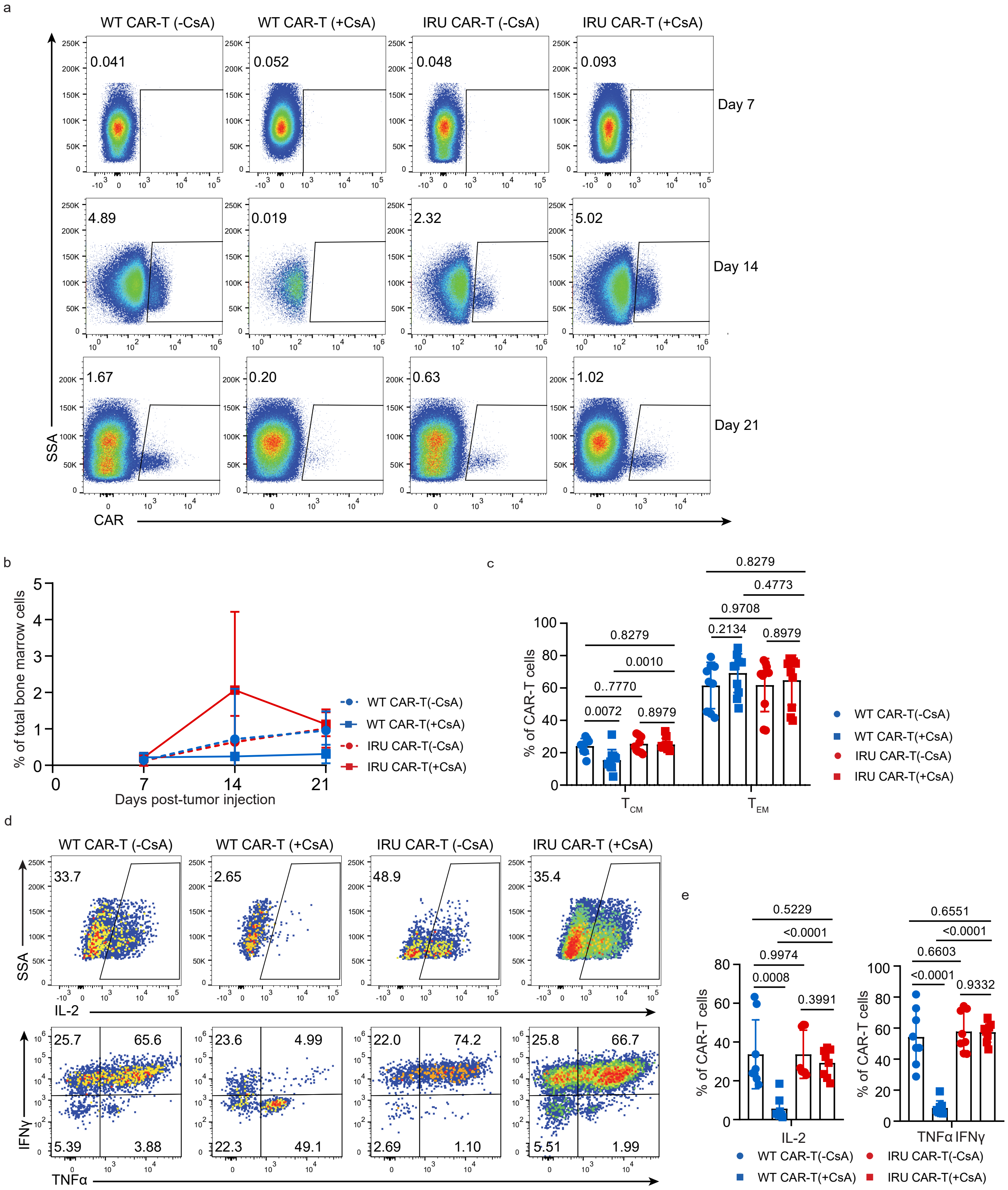

Supplementary Fig. 7 | IRU CAR-T cells exerted anti-tumor functions in mouse model and their *ex vivo* status. a-e, in ex vivo assays, mice in each group were euthanized at day 7, 14 and 21. CAR-T cells in bone marrow were analyzed by FACS. **a-c**, Representative flow plots (**a**) and curve (**b**) of CAR expression at different time points (n=8, 14 and 7 animals at day 7, 14 and 21 respectively in WT CAR-T (-CsA); n=8, 24 and 8 animals at day 7, 14 and 21 respectively in WT CAR-T (+CsA); n=8, 15 and 8 animals at day 7, 14 and 21 respectively in IRU CAR-T (-CsA); n=8, 17 and 8 animals at day 7, 14 and 21 respectively in IRU CAR-T (+CsA)). **c**, the differentiation of IRU CAR-T cells at day 14 (n=9 animals in WT CAR-T (-CsA); n=11 animals in WT CAR-T (-CsA); n=10 animals in IRU CAR-T (-/+CsA);). **d-e**, Representative flow plots (**d**) and column charts (**e**) showed the cytokine production of IRU CAR-T cells at day 14 (n=8 animals in each group). At least two batches were performed, and indicated in Fig. 3. All data are means±s.d. P values were determined by multiple t test (**c**,**e**). WT CAR-T (-CsA) as blue circle, WT CAR-T (+CsA) as blue square, IRU CAR-T (-CsA) as red circle and IRU CAR-T (+CsA) as red square.

a

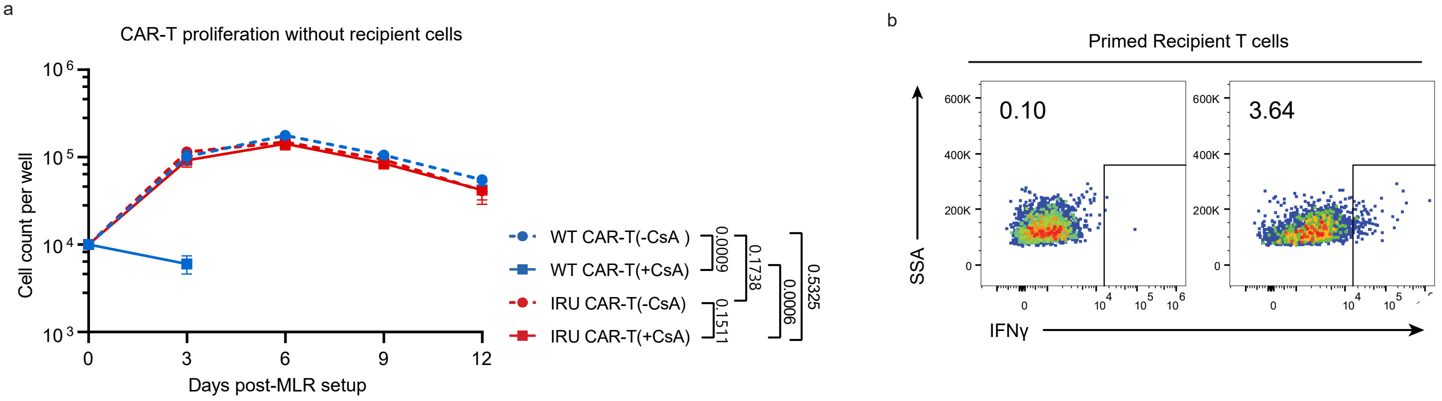

Supplementary Fig. 8 | IRU CAR-T cells can proliferate in the presence of CsA and RTCs can produce IFNγ against CAR-T cells after primed by donor PBMCs. a, CAR-T proliferation without recipient T cells (n=3 biologically independent samples). **b,** The IFNγ producing cell percentage of RTCs after primed by donor PBMCs for 6 days . Left: RTCs that were not primed by donor PBMCs. Right: RTCs that were primed by donor PBMCs. All data are means±s.d. P values were determined by Multiple t test adjusted by the Holm-Sidak method (**a**). WT CAR-T (-CsA) as blue circle, WT CAR-T (+CsA) as blue square, IRU CAR-T (-CsA) as red circle and IRU CAR-T (+CsA) as red square.

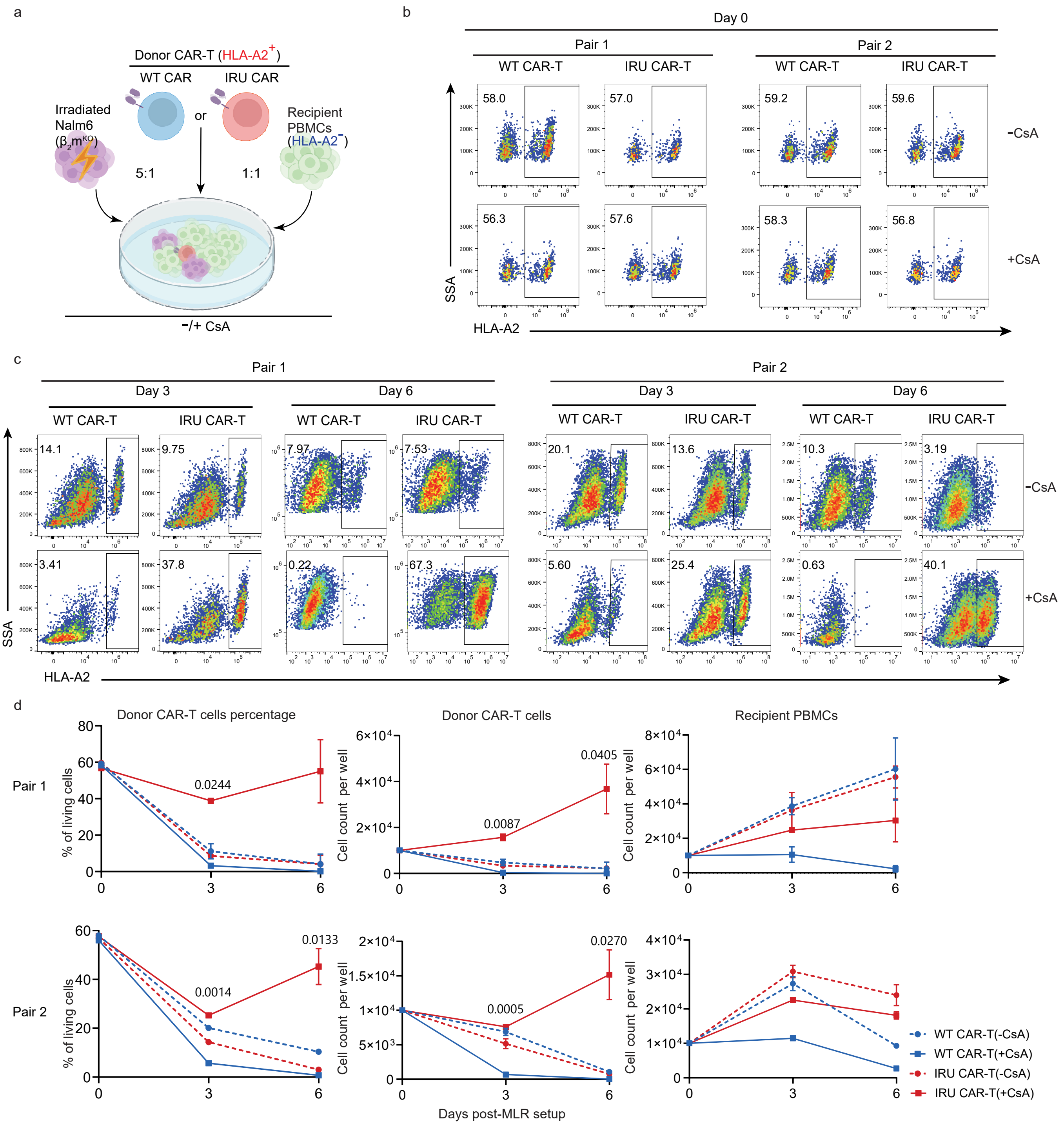

Supplementary Fig. 9 | IRU CAR-T cells retained effector functions in the presence of allogeneic cells *in vitro* in different donor-recipient pairs. a, Schematic of MLR-PBMCs for **b-d** (n = 2 technical replicates per group). Two pairs of donor CAR-T (WT/IRU CAR; HLA-A2+) and 10-fold recipient PBMCs (HLA-A2-) were mixed respectively upon the stimulation of 5-fold irradiated (50 Gy) Nalm6-FFluc-GFP in the presence/absence of CsA. **b-c,** Representative flow plots (**b, c**) and a curve chart showing percentage of donor CAR-T cells (**d**, left) on day 0, 3 and 6, and curve charts of absolute cell counts of donor CAR-T cells (**d**, middle) and recipient PBMCs (**d**, right). All data are means±s.d. P values were determined by Multiple t test adjusted by the Holm-Sidak method (**d**). WT CAR-T (-CsA) as blue circle, WT CAR-T (+CsA) as blue square, IRU CAR-T (-CsA) as red circle and IRU CAR-T (+CsA) as red square.

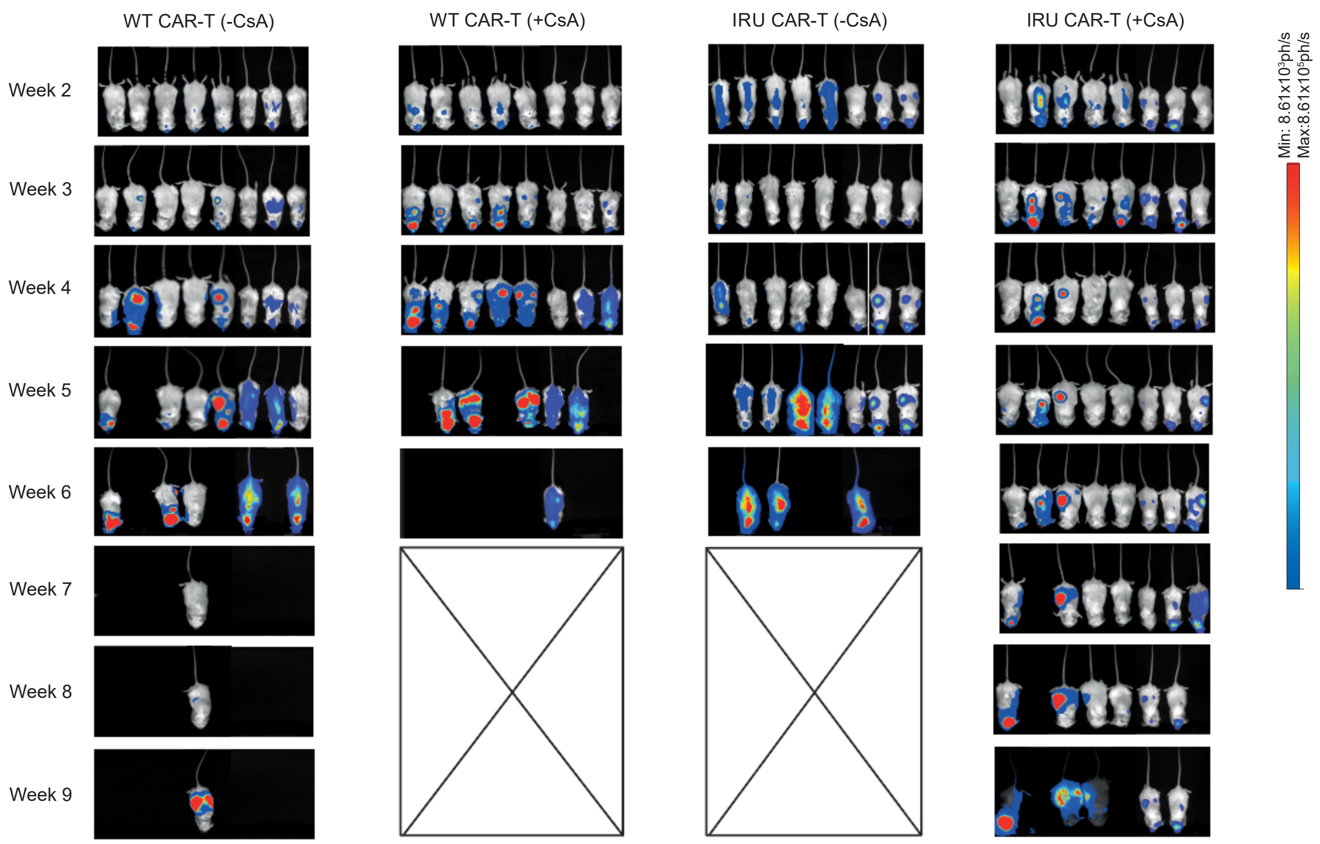

Supplementary Fig. 10 | Light emission detection showed the tumour progression in mouse systemic T-cell-mediated rejection tumor model (n=8 animals in each group, 2 batches).

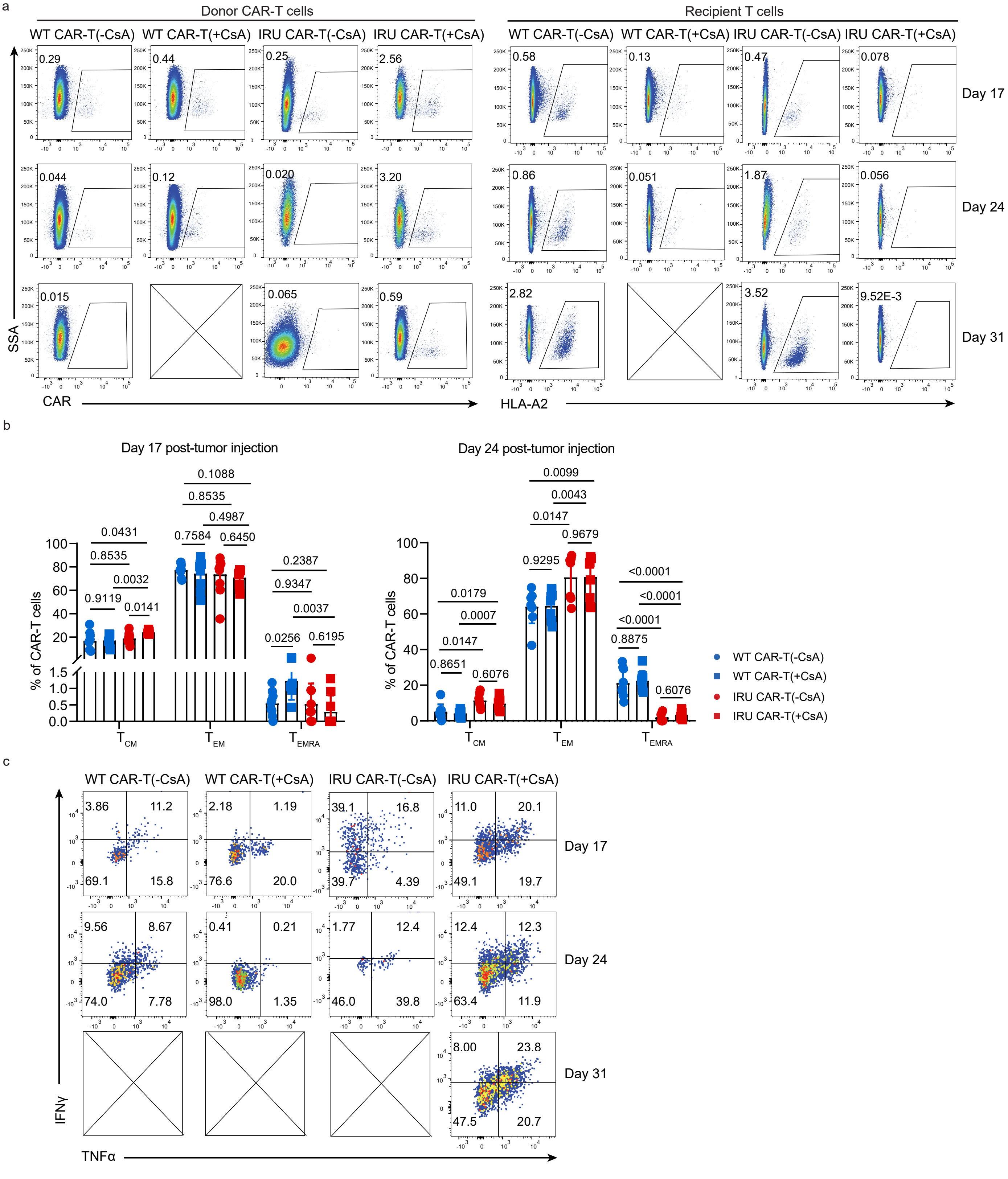

Supplementary Fig. 11 | IRU CAR-T cells retained efficacies in mouse systemic T-cell-mediated rejection tumor model and *ex vivo* analysis of IRU CAR-T cells in mouse systemic T-cell-mediated rejection tumor model. In *ex vivo* assays, Mice in each group were euthanized at day17, 24 and 31. CAR-T and RTCs in bone marrow cells were analyzed by FACS. **a,** Representative flow plots of CAR (**a**, left) and RTCs (HLA-A2$^+$, **a**, right) expression at day 17, 24 and 31. **b,** Phenotype of CAR-T cells (n=9 animals in each group). **c,** Representative flow plots of cytokine production of CAR-T cells in mouse systemic T-cell-mediated rejection tumor model. All data are means±s.d. P values were determined by Multiple t test adjusted by the Holm-Sidak method (**b**). WT CAR-T (-CsA) as blue circle, WT CAR-T (+CsA) as blue square, IRU CAR-T (-CsA) as red circle and IRU CAR-T (+CsA) as red square.

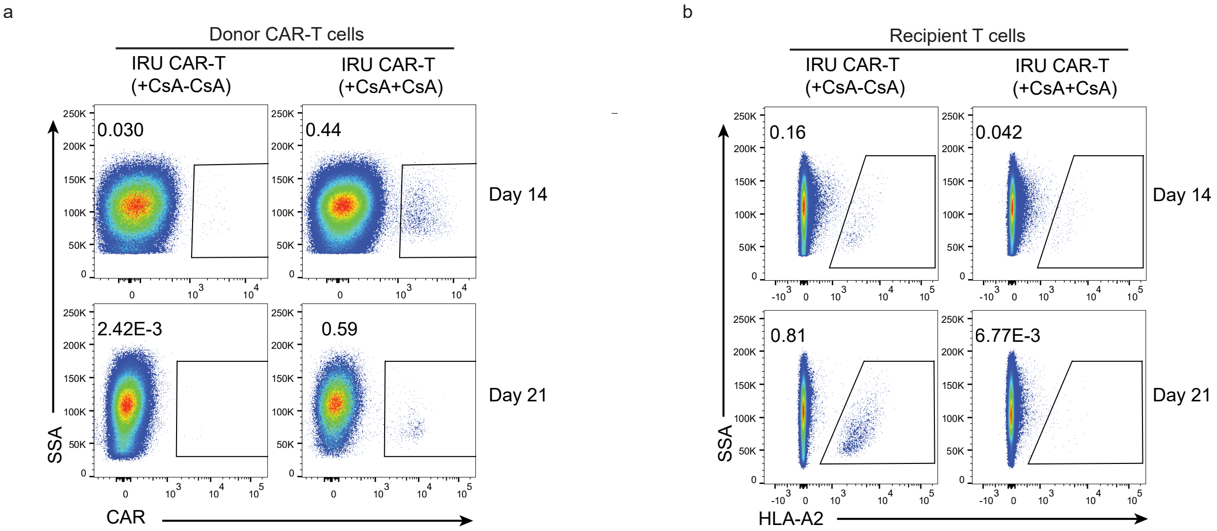

Supplementary Fig. 12 | *Ex vivo* analysis of IRU CAR-T cells in mouse systemic T-cell-mediated re-challenge rejection tumor model. Representative flow plots of CAR (**a**) and RTCs (HLA-A2$^+$, **b**) expression at day 14 and 21.

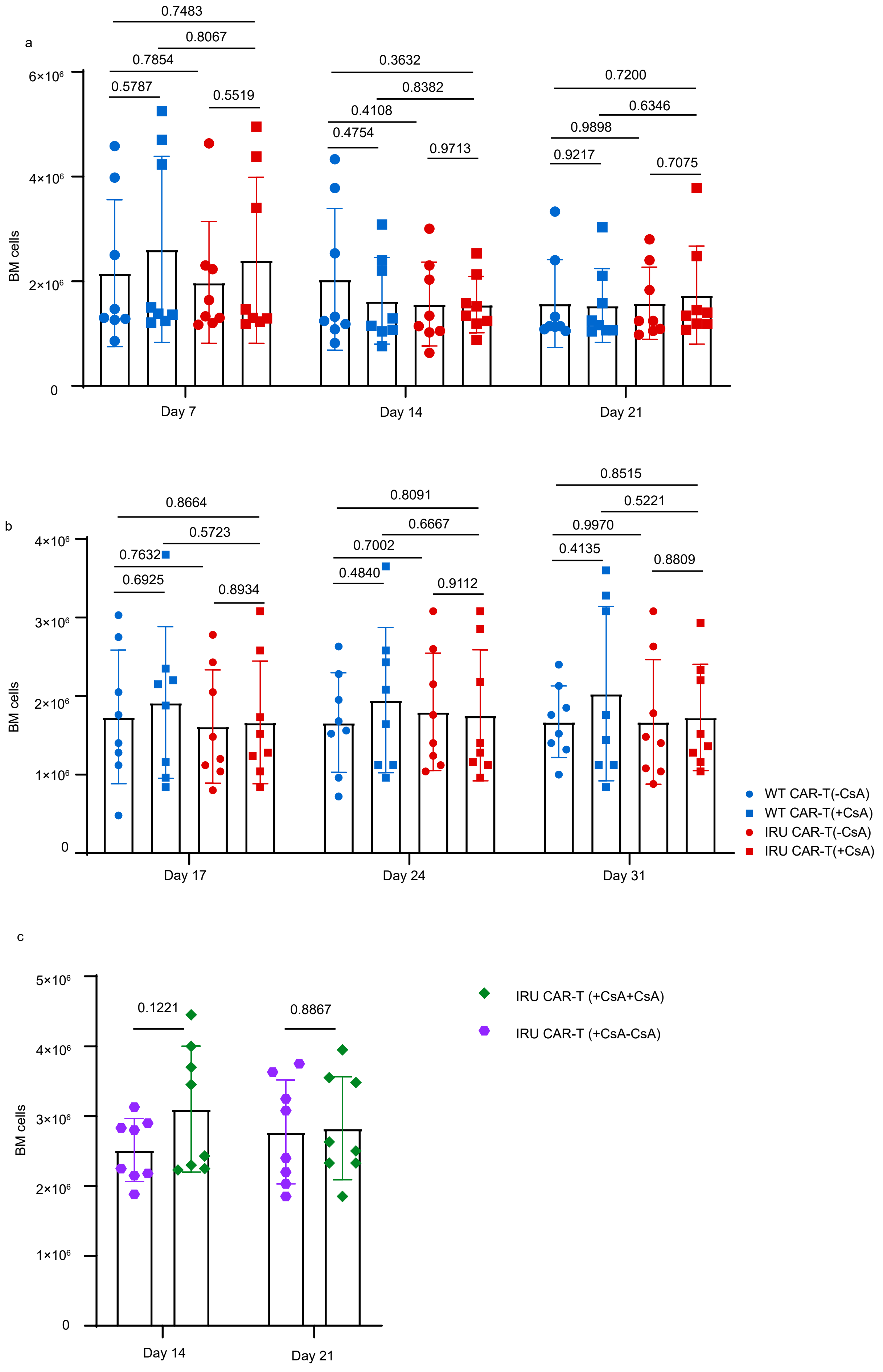

Supplementary Fig. 13 | Bone marrow cells count in mouse models. a, Bone marrow cells in ALL mouse models. **b,** Mouse ALL model with RTCs. **c,** Mouse ALL model with rechallenged RTCs. WT CAR-T (-CsA) as blue circle, WT CAR-T (+CsA) as blue square, IRU CAR-T (-CsA) as red circle, IRU CAR-T (+CsA) as red square, IRU CAR-T (+CsA+CsA) as green diamond, and IRU CAR-T (+CsA-CsA) as purple hexagon. P values were determined by multiple t test adjusted by the Holm-Sidak method.

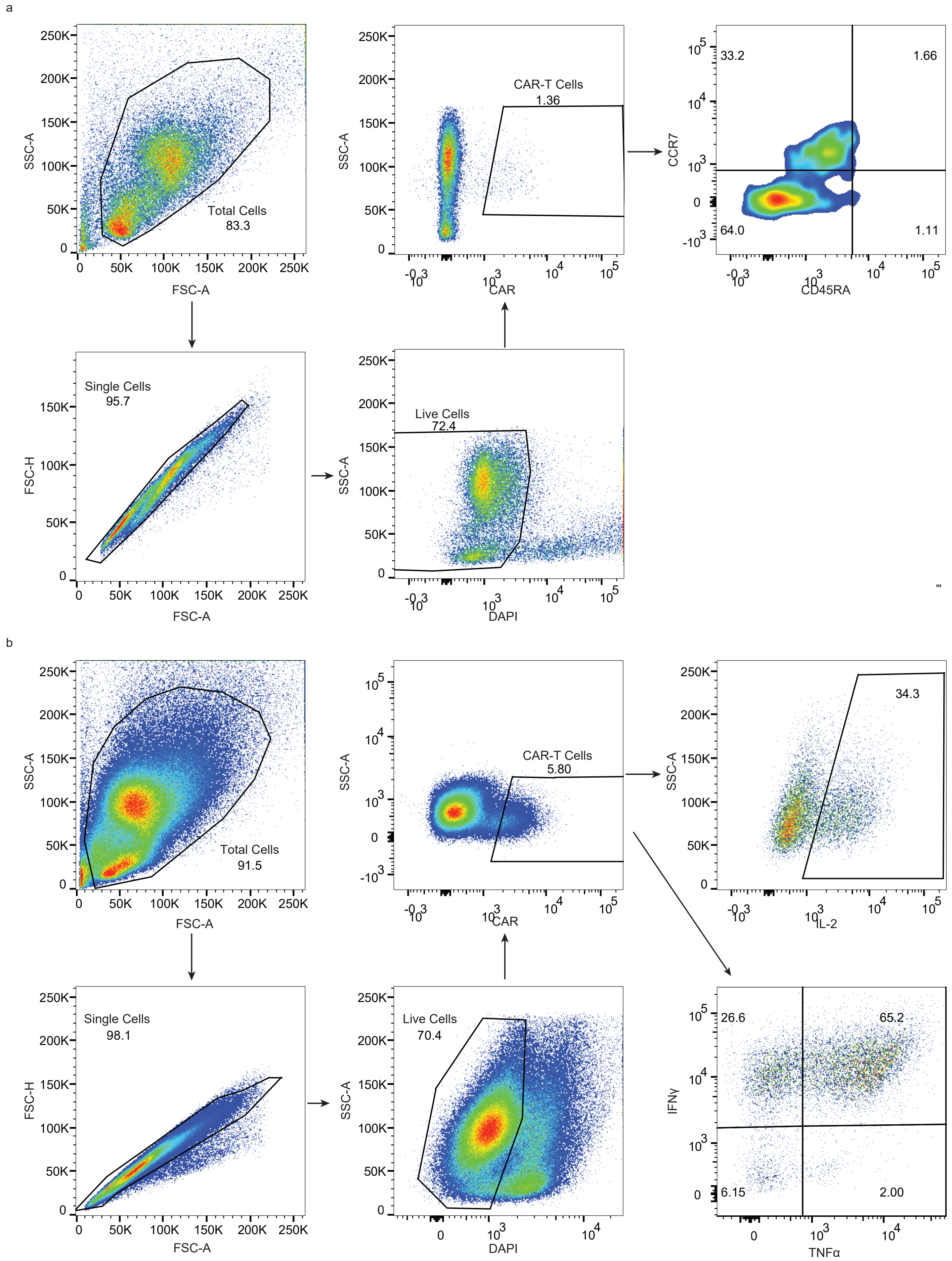

Supplementary Fig. 14 | Gating strategy in mouse ALL model. a,b, Representative flow cytometric analysis of phenotype of CAR-T cells in bone marrow (**a**) and cytokines of CAR-T cells after stimulation with PMA/Ionomycin for 4 hrs (**b**). Placement of gating was based on FMO controls.

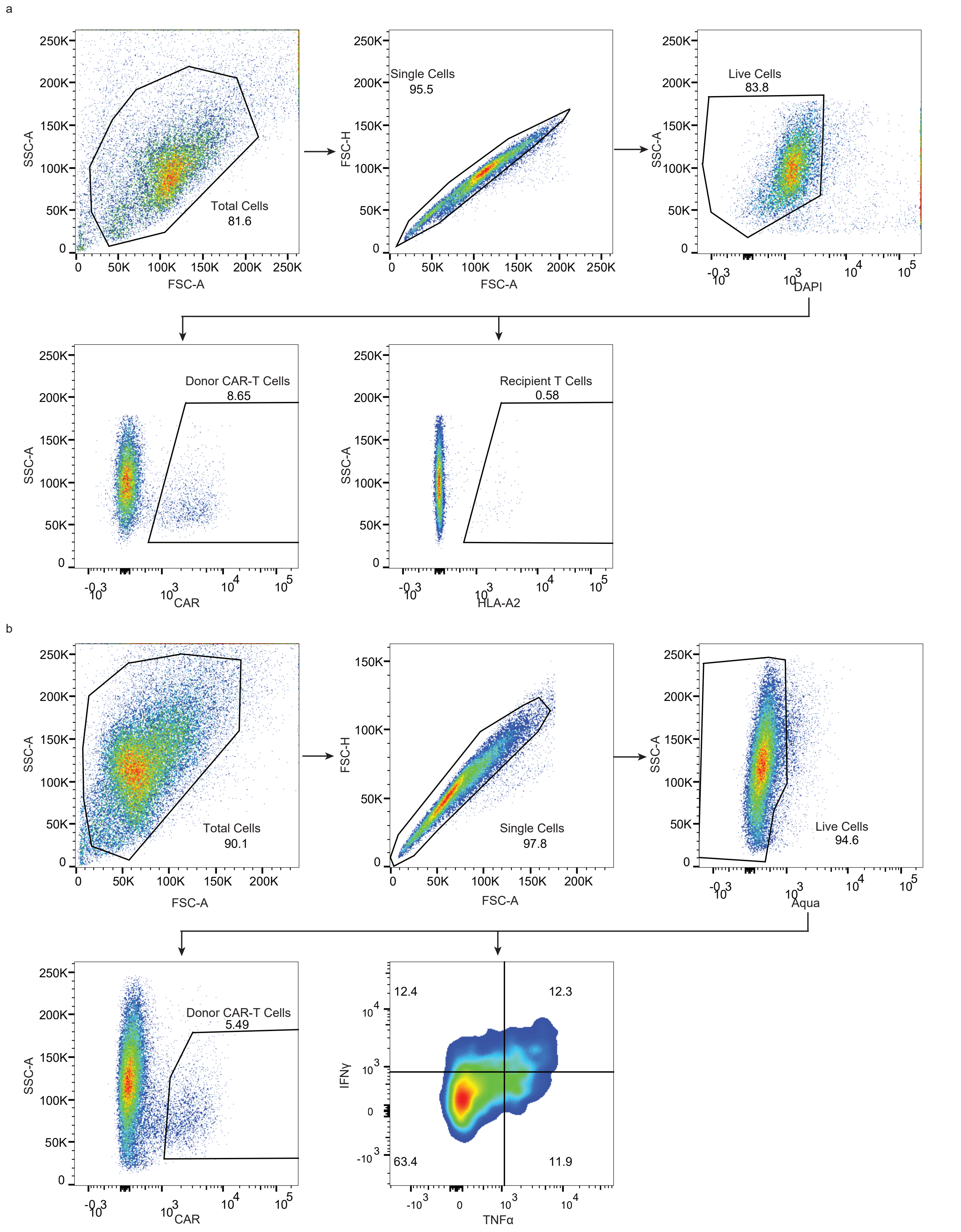

Supplementary Fig. 15 | Gating strategy in mouse ALL model with RTCs. a,b, Representative flow cytometric analysis of donor CAR-T and recipient T cells in bone marrow (**a**) and cytokines of donor CAR-T cells after stimulation with PMA/Ionomycin for 4 hrs (**b**). Placement of gating was based on FMO controls.

Table 1: Primers for qPCR.

ID	Primer Name	Primer sequence (5'to3')
1	IL2-F	AGAACTCAAACCTCTGGAGGAAG
2	IL2-R	GCTGTCTCATCAGCATATTCACAC
3	IFNG-F	GAGTGTGGAGACCATCAAGGAAG
4	IFNG-R	TGCTTTGCGTTGGACATTCAAGTC
5	TNF-F	CTCTTCTGCCTGCTGCACTTTG
6	TNF-R	ATGGGCTACAGGCTTGTCACTC
7	IL5-F	GGAATAGGCACACTGGAGAGTC
8	IL5-R	CTCTCCGTCTTTCTTCTCCACAC
9	IL3-F	AAGCAGCCACCTTTGCCTTTGC
10	IL3-R	ACAGCCCTGTTGAATGCCTCCA
11	CDC25A-F	TCTGGACAGCTCCTCTCGTCAT
12	CDC25A-R	ACTTCCAGGTGGAGACTCCTCT
13	ATM-F	TGTTCCAGGACACGAAGGGAGA
14	ATM-R	CAGGGTTCTCAGCACTATGGGA
15	NTRK1-F	CACTAACAGCACATCTGGAGACC
16	NTRK1-R	TGAGCACAAGGAGCAGCGTAGA
17	CDK4-F	CCATCAGCACAGTTCGTGAGGT
18	CDK4-R	TCAGTTCGGGATGTGGCACAGA
19	CSF2-F	GGAGCATGTGAATGCCATCCAG
20	CSF2-R	CTGGAGGTCAAACATTTCTGAGAT
21	GZMB-F	CGACAGTACCATTGAGTTGTGCG
22	GZMB-R	TTCGTCCATAGGAGACAATGCCC
23	B2M-F	CCACTGAAAAAGATGAGTATGCCT
24	B2M-R	CCAATCCAAATGCGGCATCTTCA
