# Supplementary material for: Cyclosporine A-resistant CAR-T cells mediate antitumour immunity in the presence of allogeneic cells
Source: Nat Commun. 2023 Dec 20;14:8491. doi: 10.1038/s41467-023-44176-0 (PMC10733396; doi:10.1038/s41467-023-44176-0)
Supplement: Supplementary file 3 — Reporting Summary [file 41467_2023_44176_MOESM3_ESM.pdf]

nature portfolio

Corresponding author(s): Xun Zeng

Last updated by author(s): Jul 25, 2023

Reporting Summary

Nature Portfolio wishes to improve the reproducibility of the work that we publish. This form provides structure for consistency and transparency in reporting. For further information on Nature Portfolio policies, see our Editorial Policies and the Editorial Policy Checklist.

Statistics

For all statistical analyses, confirm that the following items are present in the figure legend, table legend, main text, or Methods section.

n/a	Confirmed	
☐	☒	The exact sample size (*n*) for each experimental group/condition, given as a discrete number and unit of measurement
☐	☒	A statement on whether measurements were taken from distinct samples or whether the same sample was measured repeatedly
☐	☒	The statistical test(s) used AND whether they are one- or two-sided<br>*Only common tests should be described solely by name; describe more complex techniques in the Methods section.*
☒	☐	A description of all covariates tested
☒	☐	A description of any assumptions or corrections, such as tests of normality and adjustment for multiple comparisons
☐	☒	A full description of the statistical parameters including central tendency (e.g. means) or other basic estimates (e.g. regression coefficient) AND variation (e.g. standard deviation) or associated estimates of uncertainty (e.g. confidence intervals)
☐	☒	For null hypothesis testing, the test statistic (e.g. *F*, *t*, *r*) with confidence intervals, effect sizes, degrees of freedom and *P* value noted<br>*Give P values as exact values whenever suitable.*
☒	☐	For Bayesian analysis, information on the choice of priors and Markov chain Monte Carlo settings
☒	☐	For hierarchical and complex designs, identification of the appropriate level for tests and full reporting of outcomes
☒	☐	Estimates of effect sizes (e.g. Cohen's *d*, Pearson's *r*), indicating how they were calculated

Our web collection on statistics for biologists contains articles on many of the points above.

Software and code

Policy information about availability of computer code

Data collection	For flow cytometry: LSRFortessa (BD Biosciences) or a CytoFLEX or DxFLEX flow cytometer (BECKMAN COULTER);<br>For in vivo imaging: biospace lab photon image optima
Data analysis	For flow cytometry: FlowJo v.10 software; For in vivo imaging: M3Vision (v1.1.3); For data analysis: GraphPad Prism 10; For RNA-seq: STAR (v.2.7.2a), DEseq2 (v1.34.0), ClusterProfiler (v4.2.2), and R package pheatmap (1.0.12).

For manuscripts utilizing custom algorithms or software that are central to the research but not yet described in published literature, software must be made available to editors and reviewers. We strongly encourage code deposition in a community repository (e.g. GitHub). See the Nature Portfolio guidelines for submitting code & software for further information.

Data

Policy information about availability of data

All manuscripts must include a data availability statement. This statement should provide the following information, where applicable:

- Accession codes, unique identifiers, or web links for publicly available datasets
- A description of any restrictions on data availability
- For clinical datasets or third party data, please ensure that the statement adheres to our policy

The RNA-seq data has been deposited in the Sequence Read Archive (SRA) under accession code PRJNA880631 (https://www.ncbi.nlm.nih.gov/bioproject/PRJNA880631). The raw data generated in this study is provided in the Source Data file.

Human research participants

Policy information about studies involving human research participants and Sex and Gender in Research.

Reporting on sex and gender	Sex: female or male. Consents have been obtained.
Population characteristics	Volunteers are Asian people in the age range of 25-46, female and male.
Recruitment	Healthy participants were recruited through public recruitment advertisement which was approved by the First Affiliated Hospital, College of Medicine, Zhejiang University. Fresh PBMCs from healthy donors were provided by the First Affiliated Hospital, College of Medicine, Zhejiang University. The recruitments of healthy human blood donors were approved by the Clinical Research Ethics Committee of the First Affiliated Hospital, College of Medicine, Zhejiang University. All the participants signed the Informed Consent Form.
Ethics oversight	All experiments conducted on human material were approved by the Clinical Research Ethics Committee of the First Affiliated Hospital, Zhejiang University School of Medicine in 2022, No. 1013-Quick.

Note that full information on the approval of the study protocol must also be provided in the manuscript.

Field-specific reporting

Please select the one below that is the best fit for your research. If you are not sure, read the appropriate sections before making your selection.

[x] Life sciences [ ] Behavioural & social sciences [ ] Ecological, evolutionary & environmental sciences

For a reference copy of the document with all sections, see nature.com/documents/nr-reporting-summary-flat.pdf

Life sciences study design

All studies must disclose on these points even when the disclosure is negative.

Sample size	No statistical methods were used to pre-determine sample size. Sample sizes were estimated based on preliminary experiments, with an effort to achieve a minimum of n=5 mice per treatment group unless specified.
Data exclusions	No data were excluded throughout the studies.
Replication	All experiments were repeated with similar results as shown in figures and stated in the figure legends.
Randomization	No mice were excluded. Mice were randomly assigned into control or treatment groups by an operator who was blinded to the experiments.
Blinding	Mouse condition and survival were observed by an operator who was blinded to treatment groups in addition to the main investigator who was not blind to group allocation. Tumor burden and other data analyses are based on objectively measurable data (e.g. fluorescence intensity). As the outcomes of survival and tumor burden were objective, the involvement of one investigator who was not blind to group allocation would not influence the interpretation of outcome.

Reporting for specific materials, systems and methods

We require information from authors about some types of materials, experimental systems and methods used in many studies. Here, indicate whether each material, system or method listed is relevant to your study. If you are not sure if a list item applies to your research, read the appropriate section before selecting a response.

Materials & experimental systems

n/a	Involved in the study	
[ ]	[x]	Antibodies
[ ]	[x]	Eukaryotic cell lines
[x]	[ ]	Palaeontology and archaeology
[ ]	[x]	Animals and other organisms
[x]	[ ]	Clinical data
[x]	[ ]	Dual use research of concern

Methods

n/a	Involved in the study	
[x]	[ ]	ChIP-seq
[ ]	[x]	Flow cytometry
[x]	[ ]	MRI-based neuroimaging

Antibodies

Antibodies used	Alexa Fluor 647-Rabbit Anti-Mouse FMC63 scFv Polyclonal Antibody (BIOSWAN, Clone: R19M; Cat: 200102; Lot: 210203.); Brilliant Violet 510-HLA-A2 (Biolegend, Clone: BB7.2; Cat: 343320; Lot: B314487); Brilliant Violet 510-CD3ε (Biolegend, Clone: OKT3; Cat: 317332; Lot: B333191); PE- CD3ε (Biolegend, Clone: OKT3; Cat: 300308; Lot: B314341); Brilliant Violet 605-CD4 (Biolegend, Clone: SK3; Cat: 344646; Lot: B359404); Brilliant Violet 711-CD8 (Biolegend, Clone: SK1; Cat: 344734; Lot: B360808); APC/Cyanine7-CD8 (Biolegend, Clone: SK1; Cat: 344714; Lot: B325254); APC-CD19 (Biolegend, Clone: HIB19, Cat: 302212; Lot: B363120); PerCP/Cyanine5.5-CD45RA (Biolegend, Clone: HI100; Cat: 304122; Lot: B348765); Alexa Fluor 700-CCR7 (Biolegend, Clone: G043H7; Cat: 353244; Lot: B347206); Pacific Blue-IL-2 (Biolegend, Clone: MQ1-17H12; Cat: 500324; Lot: B317866); PE-IFNγ (Biolegend, Clone: 4S.B3; Cat: 502509; Lot: B352970); PerCP/Cyanine5.5-TNFα (Biolegend, Clone: MAb11; Cat: 502926; Lot:B339549). Alexa Fluor® 647 anti-HA.11 Epitope Tag Antibody (Biolegend, Clone: 16B12; Cat: 682404; Lot: B353930). Goat anti-Rabbit IgG (H+L) Highly Cross-Adsorbed Secondary Antibody, Alexa Fluor™ 568, Invitrogen™ (Invitrogen, Cat: A11036; Lot: 2045347). Recombinant Anti-NFAT2 (phospho S237) antibody (Abcam, Cat: ab183023; Lot: 1003619-1)
Validation	All antibodies used in this study are commercially available. Antibody validations were performed by the suppliers and the information is provided on the website and product information datasheets. The certificate of analysis (CoA) was provided for the quality assurance of each antibody lot.

Eukaryotic cell lines

Policy information about cell lines and Sex and Gender in Research

Cell line source(s)	Nalm6-FFluc-GFP was from Shanghai Model Organisms. Jurkat-NFAT-GFP cells were a kind gift from prof. Wei Chen, Zhejiang University. HepG2 cells were a kind gift from prof. Jie Sun, Zhejiang University (originally purchased from Procell, Cat: SL0103). AAV-293 cells were obtained from the Cell Line Ontology Subset for Chinese National Infrastructure. Primary cells were donated from healthy volunteers.
Authentication	Authentication procedures were provided with cell line by their original providers, with COA provided. Properties pertinent to the experiments (GFP and CD19 expression for Nalm6-FFluc-GFP cells, GFP expression for Jurkat-NFAT-GFP cells and HepG2) were further confirmed by flow cytometry in preliminary experiments.
Mycoplasma contamination	All cell lines were routinely tested for mycoplasma and were found to be negative.
Commonly misidentified lines (See ICLAC register)	No commonly misidentified cell lines were used.

Animals and other research organisms

Policy information about studies involving animals; ARRIVE guidelines recommended for reporting animal research, and Sex and Gender in Research

Laboratory animals	NOD.Cg-PrkdcscidIl2rgem1Smoc (NSG) (female, aged 8-12 weeks) were purchased from the Shanghai Model Organisms Center, Inc. (Stock: NM-NSG-001) Shanghai Model Organisms and bred in the Laboratory Animal Center Hangzhou Normal University (specific pathogen-free level). Other parameters included: dark/light cycle 12/12 (6:00-18:00 light), ambient temperature 21-23oC, and humidity 40-70%.
Wild animals	No wild animals were used in the study.
Reporting on sex	The disease model and mice performance are sex-independent. Female mice were used for the convenience of mice operation, maintaining and experiments.
Field-collected samples	No field collected samples were used in the study.
Ethics oversight	All animal experiments were conducted in compliance with relevant animal use guidelines and ethical regulations, and approved by Ethics Committee of Hangzhou Normal University No. HSD20210703.

Note that full information on the approval of the study protocol must also be provided in the manuscript.

Flow Cytometry

Plots

Confirm that:

- [x] The axis labels state the marker and fluorochrome used (e.g. CD4-FITC).
- [x] The axis scales are clearly visible. Include numbers along axes only for bottom left plot of group (a 'group' is an analysis of identical markers).
- [x] All plots are contour plots with outliers or pseudocolor plots.
- [x] A numerical value for number of cells or percentage (with statistics) is provided.

Methodology

Sample preparation	Buffy coats from healthy donors and peripheral blood from healthy volunteers were isolated and purified as described in Methods. Bone marrow cells of mice were obtained from hind limb. Muscles surrounding the limb were removed and bones were crushed in a mortar with addition of FACS buffer, passed through a cell strainer and collected in a tube. Cells were pelleted and red blood cells were lysed with the addition of ACK lysis buffer and subsequently washed. Cells were subsequently washed, resuspended in FACS Buffer with Fc blocking reagent; antibodies were added and washed off after the incubation time. If intracellular staining was performed, cells were fixed and permeabilized using the fixation buffer (Biolegend) according to the manufacturer's instructions. For analysis of live cells, DAPI (ThermoFisher) and Aqua (Biolegend) were used as viability dyes.
Instrument	LSRFortessa (BD Biosciences) or a CytoFLEX or DxFLEX flow cytometer (BECKMAN COULTER);
Software	FlowJo v.10 software
Cell population abundance	The purity was verified by flow cytometry.
Gating strategy	The starting cell population was gated on a SSC-A/FSC-A plot. Cell singlets were identified by FSC-A/FSC-H gating. Positive/ Negative populations were determined by ISO controls.

- [x] Tick this box to confirm that a figure exemplifying the gating strategy is provided in the Supplementary Information.
